# Gene expression and promoter methylation of porcine uncoupling protein 3 gene

**DOI:** 10.5713/ajas.18.0116

**Published:** 2018-07-26

**Authors:** Ruiyi Lin, Weimin Lin, Qiaohui Chen, Jianchao Huo, Yuping Hu, Junxiao Ye, Jingya Xu, Tianfang Xiao

**Affiliations:** 1 College of Animal Science, Fujian Agriculture and Forestry University, Fuzhou, Fujian 350002, China

**Keywords:** Pig, Uncoupling Protein 3 (*UCP3*) Gene, Meat Quality, Methylation, Expression

## Abstract

**Objective:**

Uncoupling protein 3 gene (*UCP3*) is a candidate gene associated with the meat quality of pigs. The aim of this study was to explore the regulation mechanism of *UCP3* expression and provide a theoretical basis for the research of the function of porcine *UCP3* gene in meat quality.

**Methods:**

Bisulfite sequencing polymerase chain reaction (PCR) and quantitative real-time PCR (Q-PCR) were used to analyze the methylation of *UCP3* 5′-flanking region and *UCP3* mRNA expression in the adipose tissue or skeletal muscle of three pig breeds at different ages (1, 90, 210-day-old Putian Black pig; 90-day-old Duroc; and 90-day-old Dupu).

**Results:**

Results showed that two cytosine-guanine dinucleotide (CpG) islands are present in the promoter region of porcine *UCP3* gene. The second CpG island located in the core promoter region contained 9 CpG sites. The methylation level of CpG island 2 was lower in the adipose tissue and skeletal muscle of 90-day-old Putian Black pigs compared with 1-day-old and 210-day-old Putian Black pigs, and the difference also existed in the skeletal muscle among the three 90-day-old pig breeds. Furthermore, the obvious changing difference of *UCP3* mRNA expression was observed in the skeletal muscle of different groups. However, the difference of methylation status and expression level of *UCP3* gene was not significant in the adipose tissue.

**Conclusion:**

Our data indicate that *UCP3* mRNA expression level was associated with the methylation status of *UCP3* promoter in the skeletal muscle of pigs.

## INTRODUCTION

With the improvements in living standards, people tend to be interested in eating high-quality pork. Improvement of porcine meat quality has become a priority when it comes to pig breeding and is also one of the ways in which breeding enterprises can earn more [1]. Uncoupling protein 3 gene (*UCP3*) was reported to wield a significant influence on fat deposition in pigs, which is an important factor affecting meat quality, especially influencing the juiciness, tenderness, and color of pork [2–4]. *UCP3* gene belongs to the mitochondrial anion carrier gene family, mainly involved in the uncoupling effect of mitochondrial respiratory chain and has an important impact on separating oxidative phosphorylation from the synthesis of ATP as energy which is anticipated as heat [5,6].

Porcine *UCP3* gene is located on chromosome 9p21-p24 with the full-length open reading frame of 927 bp, coding 308 amino acids [7,8]. The chromosome location of *UCP3* gene is consistent with the notion that numerous quantitative trait loci (QTLs) related to carcass and meat quality traits were located on SSC9 in swine (https://www.animalgenome.org/cgi-bin/QTLdb/SS/viewmap). In a study on pigs at high ambient temperature, it was reported that the *UCP3* expression was upregulated in the muscle [9]. *UCP3* mRNA expression in adipose tissue was found to be differentially associated with leptin between groups according to the birth weight of piglets [10]. In mice, the overexpression of *UCP3* protected against the accumulation of triglyceride in both muscle and adipose tissue [11]. Lin et al [12] discovered that *UCP3* could mediate heat production in cold-resistant pigs to adapt to cold environments; furthermore, eight dominant pig breeds from China could be classified into cold-sensitive and cold-resistant breeds based on the sequence of *UCP3*.

The polymorphisms of *UCP3* gene have been reported to be associated with meat quality traits in pigs. Cieslak et al [13] found a novel missense substitution in the *UCP3* and the polymorphism of *UCP3* (g.946C>T) was potentially associated with porcine abdominal fat weight. One 9-base continuous mutated site in 3′UTR of pig *UCP3* gene was screened, and the site was identified in significantly close association with the backfat thickness at the sixth and seventh rib [14]. Tu et al [15] detected three coding-region single nucleotide polymorphisms in *UCP3* gene and found that one of the mutations may have a significant effect on several carcass and meat quality traits.

However, few studies have focused on the *UCP3* 5′-flanking or promoter region, the correlation between promoter cytosine-guanine dinucleotide (CpG) island methylation status, and *UCP3* mRNA expression. In this study, bisulfite sequencing polymerase chain reaction (PCR) was used to investigate the methylation status of the *UCP3* gene promoter and quantitative real-time PCR (Q-PCR) was used to measure *UCP3* mRNA expression in the adipose tissue or skeletal muscle of three pig breeds at different ages.

## MATERIALS AND METHODS

### Animals and tissues

All animal procedures were conducted according to the Regulations for the Administration of Affairs Concerning Experimental Animals (Ministry of Science and Technology, China, revised in July 2013) and approved by the Experimental Animal Care and Use Committee of Fujian Agriculture and Forestry University (Fuzhou, Fujian, China).

Putian Black pig is a quality native breed in Fujian province and included in the list of National Livestock and Poultry Genetic Resources Protection of China. Dupu pig is a hybrid of Duroc×Putian Black pig. All pigs (1, 90, and 210-day-old Putian Black pigs; 90-day-old Duroc; and 90-day-old Dupu, n = 4 per group) included in the study were euthanized at a commercial slaughterhouse. The adipose and skeletal muscle were removed immediately, washed briefly with phosphate-buffered saline, rapidly frozen in liquid nitrogen, and stored at −80°C.

### Bioinformatic analysis

Analysis and identification of the putative promoter region in the 5′-flanking of the *UCP3* gene were performed using the following online tools: Promoter Scan (http://www-bimas.cit.nih.gov/molbio/proscan) [16], Promoter 2.0 (http://www.cbs.dtu.dk/services/Promoter/) [17], NNPP (http://www.fruitfly.org/seq_tools/promoter.html) [18] and AliBaba2.1 (http://gene-regulation.com/pub/programs/alibaba2/index.html). The CpG island predicting and primer designing were conducted by MethPrimer (http://www.urogene.org/cgi-bin/methprimer/methprimer.cgi) [19].

### Methylation detection of CpG island

Genomic DNA was extracted from the samples by standard phenol/chloroform procedure and modified using the EZ DNA Methylation-Gold Kit (ZYMO Research, Irvine, CA, USA) according to the manufacturer’s instructions.

The reaction mixture for the analysis included 20 μL of PCR product containing 100 ng genomic DNA, 2 μL of 10×PCR buffer, 150 μM dNTPs, 1 μM of each PCR primer (Table 1), 1 U Taq DNA polymerase (TaKaRa, Tokyo, Japan), and sterilized distilled water up to 20 μL. The amplifying conditions of PCR for normal DNA were 94°C for 5 min, followed by 40 cycles of 94°C for 30 s, 51°C for 30 s, 72°C for 30 s, and 72°C for 10 min. The annealing temperature used to amplify the bisulfite-treated DNA was 58°C. The PCR products were visualized by electrophoresis in 1.5% agarose gel electrophoresis and purified by MiniBEST Agarose Gel DNA Extraction Kit (TaKaRa, Japan).

The expected PCR fragment was cloned and inserted into the pMD18-T vector. The recombinant clones were used to transform Trans5α Chemically Competent Cell (TransGen Biotech, Beijing, China). The positive recombinant clones were selected on Luria-Bertani medium containing 60 μg/mL ampicillin and confirmed by PCR. Five randomly selected positive recombinant clones from each individual were sequenced through a commercial sequencing facility (Sangon, Shanghai, China). The sequencing results were analyzed using QUMA software [20].

### RNA isolation and reverse transcription

Total RNA was extracted from frozen tissues using TRIzol reagent (Invitrogen, Carlsbad, CA, USA) according to the manufacturer’s protocol. The concentration and quality of the isolated RNA were determined using Nanodrop 2000 (Thermo Fisher Scientific, Leicester, UK) and denatured gel electrophoresis. Reverse transcription polymerase chain reaction (RT-PCR) was conducted using PrimeScript RT reagent Kit with gDNA Eraser (TaKaRa, Japan), containing oligo (dT), random primers and gDNA Eraser. After treating 1 μg RNA with gDNA Eraser, the reverse transcription reaction was carried out at 37°C for 15 min, 85°C for 5 s, and 4°C for 2 min.

### Quantitative real-time polymerase chain reaction analysis

Quantitative real-time PCR (Q-PCR) was performed using an ABI Prism 7500 sequence-detection system (Applied Biosystems, Foster City, CA, USA) with SYBR Green Realtime PCR Master Mix (Toyobo, Osaka, Japan), under the manufacturer’s instructions.

The *UCP3* fragment was amplified using the primers listed in Table 1 and the *β-actin* primers were used as internal control. The primers for Q-PCR were designed by Lin et al [12] and Erkens et al [21]. All PCRs were performed in triplicate and the expression level of *UCP3* was quantified relative to the expression of *β-actin* by employing the 2^−ΔΔCt^ value method [22].

### Data analysis

Differences between groups were performed with one-way analysis of variance and least significant difference test (V19.0, SPSS Inc., Chicago, IL, USA). Methylation levels and mRNA expression were analyzed by Pearson’s correlation.

## RESULTS

### Bioinformatics analysis of the porcine UCP3 promoter

**Figure 1:**
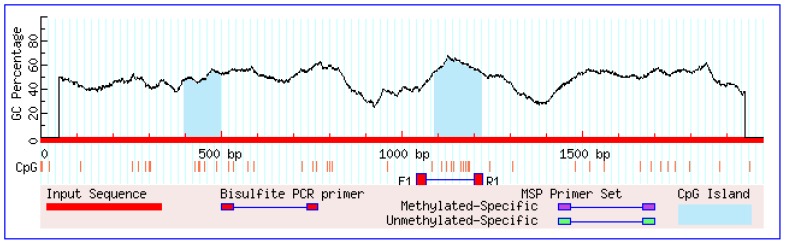
Bioinformatics analysis of the CpG island and primer design for bisulfite sequencing PCR of the porcine *UCP3* gene. The blue region is the CpG island. CpG, cytosine-guanine dinucleotide; PCR, polymerase chain reaction; *UCP3*, uncoupling protein 3.

Analysis of the porcine *UCP3* gene 5′-flanking region (2 kb upstream of the initiation codon) revealed that the core promoter region is located between nucleotides −870 and −550 and the 2 kb-fragment contains two CpG islands: −1,603 to −1,501 and −910 to −777 (Figure 1). Here, the second CpG island located within the putative promoter region was speculated to play a role in regulating gene expression. Several putative transcription factor binding sites were also identified in this CpG island, including Sp1 transcription factor, neurofibromin 1, CCAAT enhancer binding protein beta, transcription factor AP-2 alpha, CCAAT enhancer binding protein alpha, and organic cation/carnitine transporter 1. Therefore, primers were designed to amplify the fragment containing 9 CpG sites of CpG island 2.

### Methylation status of the porcine UCP3 promoter

The PCR products of normal and bisulfite-treated DNA were analyzed by using 1.5% agarose gel electrophoresis, and the positive recombinant clones were sequenced by Sangon Biotech. By comparative sequencing of the putative *UCP3* promoter region, we identified two novel single nucleotide polymorphisms (−882 A/T, −852 G/A) in the 5′-flanking region of porcine *UCP3*.

**Figure 2:**
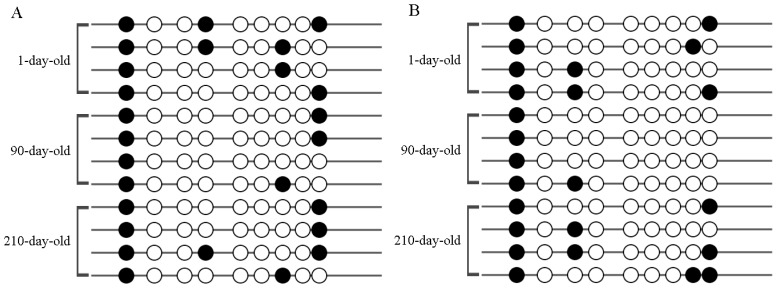
The methylation pattern of CpG island 2 of each Putian Black pig at different ages. (A) Adipose tissue, (B) skeletal muscle. Every circle represents a CG, black circles mean methylated CG, and white circles mean unmethylated CG. CpG and CG, cytosine-guanine dinucleotide.

The methylation status of the putative *UCP3* promoter region was examined in the adipose tissue and skeletal muscle of Putian Black pig at three different ages (Figure 2). Data in Table 2 showed that the degree of methylation in 90-day-old Putian Black pig was lower compared with the other two ages in skeletal muscle and adipose tissue. However, the changing of methylation status did not reach the statistical significance level in adipose tissue. Furthermore, we measured the methylation level of skeletal muscle in other two 90-day-old pig breeds (Duroc, 19.44%±5.56%, Dupu, 11.11%, Supplementary Figure S1). The methylation level in Dupu was lower than that in Duroc (p<0.05), and there was no significant difference between that of Dupu and Putian Black pig.

### mRNA expression level of *UCP3* gene

**Figure 3:**
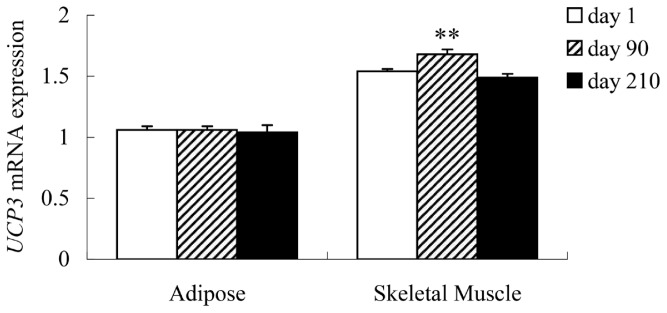
Differentiation of *UCP3* mRNA expression in adipose tissue and skeletal muscle between different ages. Values are means with their standard errors. Significant differences between groups in the same tissue are denoted by ** (p<0.01). *UCP3*, uncoupling protein 3.

**Figure 4:**
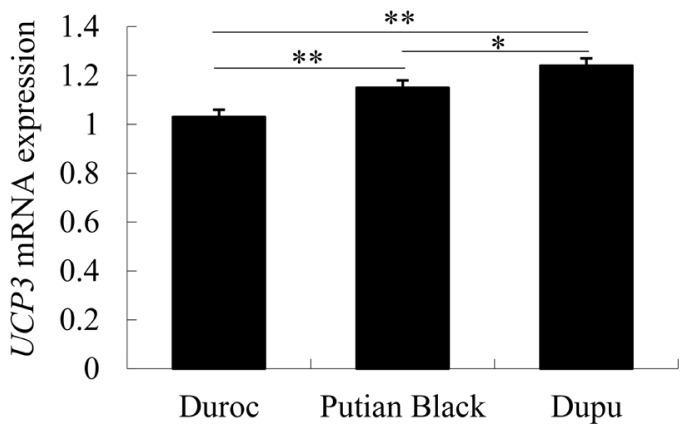
Differentiation of *UCP3* mRNA expression in skeletal muscle between different pig breeds. Values are means with their standard errors. Significant differences between breeds are denoted by * p< 0.05), ** p<0.01. *UCP3*, uncoupling protein 3.

The relative expression level of *UCP3* in the skeletal muscle of the Putian Black pig increased to the highest at 90 days (p< 0.01) and then decreased at 210 days (Figure 3). However, the *UCP3* mRNA expression level was not different in the adipose tissue of different ages. Moreover, the expression level of *UCP3* in skeletal muscle was the highest in Dupu pig and the lowest in Duroc at 90-day-old (Figure 4). Pearson’s correlation analysis showed that the methylation status of the CpG island correlated negatively with *UCP3* expression in skeletal muscle (r = −0.82 or −0.72; p<0.01); with significant correlation coefficients for CpG_9 in Putian Black pig at three stages (r = −0.632; p<0.05).

## DISCUSSION

It is well known that *UCP3* is an important candidate gene for regulating intramuscular fat to affect meat quality traits [13,14]. Putian Black pig, which is one of the native breeds in Fujian province, is famous for its meat fragrance and taste. We measured the *UCP3* promoter methylation and mRNA expression in the adipose tissue and skeletal muscle of Putian Black pig at three ages. The results showed that the methylation level in the skeletal muscle of the 90-day-old Putian Black pig was lower than that of the other two ages. Even though the changing of methylation status existed in the adipose tissue, it did not reach the statistical significance level. Similarly, the *UCP3* mRNA expression level only differed in skeletal muscle at various stages. These findings were consistent with the research that *UCP3* mRNA in adipose tissue was similar between Meishan and commercial sows at different postnatal ages, and the expression in skeletal muscle was greater on day 7 in commercial sows (p<0.05) [2].

In contrast, it has been reported that *ucp3* mRNA was highly expressed in the brown adipose tissue (BAT) of rats soon after birth, decreased until three months, and recovered thereafter [23]. Pigs however, are one of the few species so far found to lack functional BAT [24], because the functional uncoupling protein 1 was lost in pigs in a genetic event [25]. We suspected that the lack of functional BAT in pigs is responsible for the undifferentiated expression of *UCP3* gene in their adipose tissue.

Furthermore, we compared the gene expression level and promoter methylation status of *UCP3* in the skeletal muscle of 90-day-old Putian Black pig, Duroc and their crossing progenies (Dupu). The methylation level in Dupu was lower than that in Duroc (p<0.05), and there was no significant difference in Dupu and Putian Black pig. The expression level of *UCP3* gene was higher in Dupu and Putian Black pig than that in Duroc. Another study also suggested that breed can affect the methylation of *UCP3* in chicken breast muscle [26].

In the previous work, we found that the pork flavor of Dupu and Putian Black was better than that of Duroc, as Dupu and Putian Black had higher concentrations of saturated and mono-unsaturated fatty acids and lower concentrations of poly-unsaturated fatty acids than Duroc [27]. Therefore, the different expression of *UCP3* gene in different pig breeds may be related to meat quality. Interestingly, two novel SNPs (−882 A/T, −852 G/A) were detected in the various pig species. Further research is needed to determine whether the allelic variations of *UCP3* gene will affect meat quality.

Studies have shown that the methylation of CpG islands in the promoter region played a vital role in gene regulation, cell differentiation, embryonic development, and so on [28–30]. In the skeletal muscle, the methylation of *UCP3* promoter experienced a dynamic process, with the development of Putian Black pig, which agreed with the fluctuating trend of *UCP3* expression. Meanwhile, breed has a significant influence on the expression of *UCP3* via the distinctive methylation status of CpG island. This indicates that methylation of the CpG island suppresses *UCP3* gene expression.

In summary, the major findings of the present study were the differential influences of porcine breed and postnatal age on CpG methylation and expression of *UCP3* gene in skeletal muscle. These results might contribute to studies on the function of *UCP3* gene in pig breeding for meat quality.

## Supplementary Data



## Figures and Tables

**Table 1:** Primer pairs used for PCR amplification

Primer name	Primer sequences (5′-3′)	Length (bp)	Tm (°C)
*UCP3* normal primer	F: AGTTTTAGTTTATGTG	426	51
	R: ACTTACCCTCAATATT		
*UCP3* BSP primer	F: TTGTGGTATATGGAGGTTTTTAGG	151	58
	R: CTTCACTCAATAAATTAAAAATCCAAC		
*UCP3* Q-PCR primer	F: AGTGGATGTGGTGAAGACCC	142	60
	R: TTCCCAAGCGCAAAAAGGAAG		
*β****-****actin* Q-PCR primer	F: TCTGGCACCACACCTTCT	114	60
	R: TGATCTGGGTCATCTTCTCAC		

PCR, polymerase chain reaction; *UCP3*, uncoupling protein 3; BSP, bisulfite sequencing PCR; Q-PCR, quantitative real-time PCR.

**Table 2:** Methylation status of the *UCP3* gene in Putian Black pig at different ages

Tissue	1 day (n = 4)	90 days (n = 4)	210 days (n = 4)
Adipose (%)	27.78±6.42	19.44±5.56	25.00±5.56
Skeletal muscle (%)	25.00±5.56^a^	13.89±5.56^b^	27.78±6.42^a^

*UCP3*, uncoupling protein 3.

Values are means±standard error.

ab Within the same rows, values with different superscripts letters differ (p<0.05).
