# Supplementary material for: Gene expression and promoter methylation of porcine uncoupling protein 3 gene
Source: Asian-Australas J Anim Sci. 2018 Jul 26;32(2):170–5. doi: 10.5713/ajas.18.0116 (PMC6325394; doi:10.5713/ajas.18.0116)
Supplement: Supplementary file 1 [file ajas-18-0116-supplmentary.pdf]

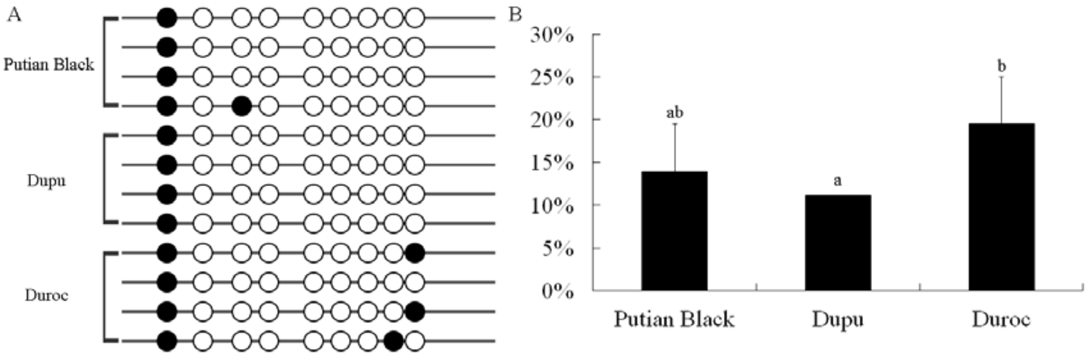

Supplementary Figure S1. Methylation levels in skeletal muscle of different pig breeds. The different lowercase letters mean different significantly ($p<0.05$).
